# An evaluation of Alberta retina health service delivery in an office setting: a cross-sectional survey of patient experience

**DOI:** 10.1186/s12913-020-05961-5

**Published:** 2020-12-04

**Authors:** Tyler Henry, Mathew Palakkamanil, Yazid N. Al Hamarneh, Matthew T. S. Tennant

**Affiliations:** Department of Ophthalmology and Visual Sciences, University of Alberta, 400, 10924 107 Avenue, Edmonton, Alberta T5H 0X5 Canada

**Keywords:** Health-care quality, Quality improvement, Ambulatory/outpatient care, Survey research and questionnaire design, Performance measurement and improvement, Quality of care, Physician learning

## Abstract

**Background:**

Retina sub-specialists provide much of the retina related eye care across Canada. In the province of Alberta, 18 retina sub-specialists work across six different offices. The purpose of this study was to assess the quality of care provided by Alberta retina sub-specialists in an office setting by administering a patient satisfaction survey. The results of this survey were provided to the same retina specialists to promote improvements in patient-centered health care delivery.

**Methods:**

A cross sectional patient satisfaction survey was performed using a thirty-part questionnaire developed in collaboration with the *Physician Learning Program* at the University of Alberta. The survey was modelled after other similar patient satisfaction surveys used in other areas of medicine. Patients from ten of the eighteen retina practices in Alberta participated in this survey. Topics of the survey included pre-appointment experience, physician-patient interactions and quality, comments/ feedback and patient demographics.

**Results:**

214 randomly sampled patients completed the survey from three geographically separate office locations in Calgary and Edmonton. 90% of patients responded that their retina sub-specialist listened adequately and provided quality care in a timely manner. Patients felt that there could be improvements to accessibility to the clinic and reduced wait times, as well as in the pre-operative consent process. Including a more complete explanation of the procedure as well as the potential risks and benefits. Only 51% of patients felt that the risks of a potential surgery had been adequately explained to them. There was a statistically significant association found between overall satisfaction and lower wait times, understanding of procedural risks and time with, listening to and involving the patient in care. There were no correlations found with other demographics such as ethnicity, sex, distance traveled or age.

**Conclusions:**

This patient satisfaction survey provided valuable patient care feedback to the retina sub-specialists of Alberta. The survey results will assist this group to improve the consent process and thereby improve patient centered health care delivery. We would recommend the distribution of this survey or other similar patient satisfaction questionnaire by retina sub-specialists to their patients to improve patient centered care in their clinics.

## Background

Ophthalmology clinics in Canada are extremely busy due to the limited number of specialists serving a population of greater than 37 million people. Retinal subspecialty office-based clinics are often crowded with patients due to the repetitive nature of treatment for common vision-threatening diseases such as wet age-related macular degeneration, diabetic retinopathy, and retinal vein occlusions. The advent of intravitreal injections to treat these three diseases has increased the workload of ophthalmologists by a factor of three or more, when compared to historical treatments such as laser, as patients commonly require between seven to nine injections in the first year of treatment [1]. Indeed, it has been reported that many retina sub-specialists in Canada assess 75 patients or more per clinic day [1].

In 2012, there were 142 practicing retina sub-specialists across Canada. This represents 27% of ophthalmology sub-specialization [2]. The number of retina sub-specialists continues to grow to meet the increasing demands of the largest growing segment of the Canadian population (older adults) [3]. Currently, many retina sub-specialists in Canada work more than 10 h per clinic day delivering patient care [1].

In response to the growing clinical care demands, retina sub-specialty clinics have been forced to continually adapt and improve clinic efficiency. For example, many clinics have automatic pre-exam testing by qualified staff that includes visual acuity, intraocular pressures, and optical coherence tomography (OCT) scans to assess the retina. Furthermore, office-based procedures are arranged to maximize patient flow through the clinic. Many clinics have consolidated injections to dedicated portions of the day to improve efficiency and reduce wait-times. Such *injection clinics* might have more than 100 patients receiving intravitreal injections in a single day. In this high-volume clinical care environment, there is a risk that the quality of health care delivery might be compromised to enable the increased volume of care [4].

Prioritizing the needs and values of patients, listening to their concerns, and involving them in decision making could also enhance the quality the delivered care and improve patient outcomes. Indeed, it has been reported that the patient-physician relationships correlate with improved patient satisfaction, compliance, and outcome expectations [5]. Furthermore, patient satisfaction has been shown to be associated with improved treatment adherence in patients with diabetes [6]. Moreover, unprofessional physician behaviours have been associated with patient dissatisfaction, complaints, and lawsuits as well as adverse outcomes of care [7]. As such, in order to keep delivering the highest quality of care, clinics need to keep open feedback channels with the patients to critique the current processes and ensure the patients’ utmost satisfaction.

The purpose of this study was to evaluate the current levels of patient satisfaction with the care delivered Alberta-based retina sub-specialists and to identify the factors associated with those levels of satisfaction. This was done with the assistance of the Physician Learning Program (PLP); an *Alberta Medical Association* program dedicated to working with physicians to use evidence-based clinical information and systems for clinical quality improvement, medical education and innovative advancement of clinical practice [8].

## Methods

In 2017, the members of the Retina Society of Alberta, a collection of retina specialists practicing in Alberta, met at their annual retreat to discuss patient care. A need was identified for evaluation of physicians (retinal subspecialists) working at retinal clinics. 18 sub-specialists agreed to develop a patient satisfaction survey that would be administered to their patients while in clinic. The anonymized information would then be shared with the group to enhance the quality of the delivered care. The group approached the PLP for assistance. A cross sectional survey of patient satisfaction of retinal clinics was conducted from May 1st, 2018 to May 4th 2018.

In the same year and early 2018, a self-made retina-specific patient satisfaction survey was developed. As no validated patient satisfaction surveys specific to ophthalmology were found in the literature, a bespoke survey was developed using similar patient satisfaction surveys in other disciplines of medicine as a reference [9, 10]. The survey was composed of the following sections: patient demographics, patient’s clinic experience, interactions between patients and clinicians, and comments for improvement. Questions were designed to be specific to issues common to retina clinics, such as long wait times for appointments, lengthy clinic visits, short patient-physician interactions, and limited explanation of procedures. The time to complete the survey was limited to a maximum of 5 min, as it was felt that survey fatigue would occur at this stage, as well possible frustration from participants who want to leave clinic quickly. A draft of the survey was distributed to all 18 retina specialists across Alberta. Feedback was received and changes to the survey were performed as required. The revised survey was then piloted on 69 patients at the Alberta Retinal Clinic to check on its fitness for purpose and facilitate its refinement prior the main study (data from the pilot study were not included in the final analysis). Only minor adjustments were made. The final survey was composed of 30 questions (Table 1). The internal consistency of responses was 0.6814 (in self-reported instruments a Cronbach’s alpha of 0.6 is considered acceptable [11]).

**Table 1:** Complete questionnaire and multiple-choice options

Questions	Response Choices
1. Was this your first time at this clinic to see a retinal specialist?	Yes
	No
2. How long did you wait from the time of your referral until your visit to the specialist at this clinic?	Less than 1 week
	1–2 weeks
	2 weeks-1 month
	More than a month
3. How long did you wait until you were seen by the specialist on your visit today?	Less than 30 min
	30 min-1 h
	1–2 h
	More than 2 h
4. What is the name of the specialist you saw today?	[Physician Name]
5. What is the reason for your visit at this clinic today?	New Retinal Issue
	Follow up
	Scheduled Injection
	Other
6. a. During visit today, did your specialist clearly explain your injection plan to you?	Yes definitely
	Yes somewhat
	No not really
	No definitely not
7. b. Did your retina specialist explain the Optical Coherence Tomography (OCT) test results to you?	Yes definitely
	Yes somewhat
	No not really
	No definitely not
8. c. Do you know what the risks associated with the injection are?	Yes definitely
	Yes somewhat
	No not really
	No definitely not
9. Have you received an injection for macular degeneration?	Yes
	No
10. How long was the wait between your referral and being seen by a retinal specialist?	Less than 1 week
	1–2 weeks
	2 weeks-1 month
	More than a month
11. Did you receive the injection on the same day you were first seen by the retinal specialist?	Yes
	No
12. What medication do you receive with your injection?	Eylea
	Lucentis
	Avastin
	I don’t know
13. Did you have a retinal detachment?	Yes
	No
a) How long was the wait between the referral for the retinal detachment and when you were seen by the retinal specialist?	[insert Answer]
b) Did you have surgery on the same day as you were first seen by the retinal specialist?	Yes
	No
c) Were you referred for a retinal tear?	Yes
	No
14. Did your specialist spend enough time with you?	Yes definitely
	Yes somewhat
	No not really
	No definitely not
15. Do you feel that your specialist listened to you?	Yes definitely
	Yes somewhat
	No not really
	No definitely not
16. Did your specialist involve you in decisions about your care as much as you wanted?	Yes definitely
	Yes somewhat
	No not really
	No definitely not
17. How confident are you that you can manage your own health with the help of your specialist?	Very Confident
	Fairly Confident
	Not Very Confident
	Not Confident
18. Did your specialist provide you with informational materials (e.g. handouts) or talk to you about resources where you could find more information regarding your health?	Yes No
19. Would you have liked to receive informational handouts or a list of information resources from your specialist?	Yes
	No
a) In what form would you have liked to receive information?	A Newsletter
	An Electronic newsletter
	Web links (internet)
	Paper Handout
20. Using any number from 1 to 5, where 1 is the poorest possible care experience and 5 is the best possible care experience, please select the number would you use to rate this clinic?	1
	2
	3
	4
	5
21. Have you utilized the on-call services of the office?	Yes
	No
a) Have you called the clinic after-hours?	Yes
	No
b) Did the physician/clinic call you back in a timely fashion?	Yes
	No
c) Were you satisfied by the service provided?	Yes definitely
	Yes somewhat
	No not really
	No definitely not
22. Were you satisfied by the access to clinic information/medical advice after-hours?	Yes definitely
	Yes somewhat
	No not really
	No definitely not
23. Do you identify as....?	Female
	Male
	Other (Specify)
24. How old are you?	Under 25
	25–34
	35–44
	45–54
	55–64
	65–74
	75–85
	Older than 85
25. Do you identify as...?	White
	Asian
	First Nations
	Black/ African American
	Hispanic/Latino
	Middle Eastern
	Other
	I’d rather not say
26. What is your postal code? This helps us understand how far you had to travel for health services.	[Postal Code]
27. How did you come to your visit today? Please check all that apply	Bicycle
	Bus
	Taxi
	DATS
	on foot
	Train
	Plane
	Other
28. Did somebody have to accompany you on your visit today?	Yes
	No
29. Please provide us with feedback to help us improve future experiences at this clinic.	[Comment]
30. Complete?	Complete
	Incomplete

Initially, all retina clinics in Alberta had agreed to participate in the survey prior to its development, yet, after the survey was created and data collection was to commence, some clinics did not respond to data collection requests. Three clinics encompassing 10 retina specialists across Alberta responded and agreed to take part and were included in our study.

Survey days were selected randomly depending on surveyor availability. On these days, all the patients of the associated specialists were eligible for participation. Patients were approached after their scheduled appointment by two trained researchers (MP and NY). All patients at the clinic were eligible to participate. The researchers asked the patients if they would like to answer a survey about their satisfaction with the care they received at the clinic. They were informed that their responses would remain anonymous and would in no way affect their care. Additionally, participants understood they could withdraw consent at any time during or after the survey by contacting the researchers. Those who were interested in taking part provided verbal informed consent before answering the survey questions. Consented patients were provided an iPad to complete the survey individually. Consent was again confirmed by participants on the iPad prior to the first question. Font size was increased, in order to accommodate many patients with impaired vision. When required, assistance in reading the questions and inputting responses was provided with minimal interaction. Surveys were conducted throughout the day starting from approximately 9 AM to 4 PM, with a short break for lunch. No incentives were provided for participating in the survey. Family members were not included in the study. The researchers conducting the surveys were involved in the development of the survey and were familiar with all questions. The survey was reviewed and practiced by the researchers prior to conducting it on patients to ensure that similar messages were used for patient recruitment while they left the clinic and similar aid was provided to those participants who required assistance. All answers were confidential while patient demographics were anonymized. Information was kept in a password protected RedCAP database. Access to data was only available to researchers involved in the study.

Statistical analysis: Statistical analysis was performed using IBM SPSS Statistics® Version 26. Descriptive statistics, reliability analysis, and chi square tests were conducted. Survey responses were analyzed using descriptive statistics. Frequency (percentage) was used for categorical variables and mean (standard derivation) for continuous variables. Chi square test was used to determine the variables that significantly affected the patient’s experience rating (overall satisfaction). Cronbach’s alpha was used to test the internal consistency (the extent to which respondents answered the questions in a logical manner).

The study was approved by the the University of Alberta Research Ethics Board (Pro00070566).

## Results

### Patient population demographics

A total of 268 were approached in the period May 1st, 2018 till May 4th 2018 (from 9 AM till 4 pm every day). Of those 214 agreed to take part. More than two thirds of the participants (67%) were from Edmonton, while the other 33% were from Calgary. Older adults (≥ 65 years old) represented 72% of the participants, 58% of the participants were female and 84% identified themselves as Caucasian. For all clinics combined, patients traveled a mean of 65 km (SD 180) to reach the clinics, traveling distances up to 2047 km [12] More than half of participants (57%) presented for a scheduled procedure (retinal injections being most common), while 35% presented for a follow-up appointment and 8% presented for new retinal issue or other reason. Table 2 presents Patient Population Demographics.

**Table 2:** Patient Demographics and Sampling

	Calgary Clinics	Edmonton Clinic	Total
**Estimated average number of daily patients**	450	450	900
**Number of surveyed patients**	70	144	214
**Largest age group**	71% > 65 years old	72% > 65 years old	72% > 65 years old
**Sex**			
Female	44	80	124
Male	25	63	88
No response	1	1	2
**Ethnic identity**			
Caucasian	56	118	174
Asian, African Canadian, First Nations or other	14	26	40
Average distance travelled (min - max)	31.4 km (SD 50 km)	81 km (SD 213 km)	65 km (SD 180 km)
**Appointment Reason**			
Injection (retinal)	29	92	121
Follow up	37	38	75
New Issue or other	4	9	13

### Patient clinical experience

Participants wait times for an appointment with a specialist ranged from less than a week to 3 months. Less than a third (30%) waited less than a week, 32% waited one to 2 weeks and 28% waited 2 weeks to 1 month and 11% waiting over a month to 3 months.

When arriving at the clinic, 47% of patients waited less than 30 min to be seen by their physician, 29% of patients waited 30 min to 1 h and 23% of patients waited more than 1 h.

The majority (93%) of the patients who utilized the on-call services (14%) indicated that the clinic and/or physician responded back in a timely fashion.

Of those who did not receive informational handouts, 14% indicated that they would’ve liked to receive one, highlighting paper format as the most desired way (50%).

Around three quarters (74%) of the participants rated their experience as the “best care experience” (five out of five) and only 7% rated it as three out of five or below. Participants rated their overall experience an average of 4.63 out of 5 across all clinics.

### Physician/ patient relations

Outlining the type and risks of intravitreal injections were identified as areas for improvement, since 81% of the participants did not know what medication they received in their injection and only 51% of patients felt confident that risks were clearly outlined. Despite the perceived lack of information being provided by their physician, 83% of the participants felt confident that they could manage their retinal problem with the help of their doctor and 71% of patients said that the results of the OCT had been explained to them. Furthermore, 79% felt involved in their treatment plan. Additionally, 90% of the participants felt very listened to (as opposed to “somewhat” or “not at all”) and 90% felt that their physician spent enough time with them during their appointment.

### Variables associated with patient’s experience rating (overall satisfaction)

The variables that were associated with higher patient experience rating (overall satisfaction) were: Shorter wait times (*p* < 0.029), physician spending enough time with the patient (*p* < 0.001), physician listening to the patient (*p* < 0.001), being involved in the decision making process(*p* < 0.001), and knowing the risk of injection (*p* = 0.019). While distance travelled (> 50 km vs < 50 km), clinic, ethnicity, age, sex, reason for visit, physician provider and whether or not the physician explained the patient’s injection plan or their OCT results did not significantly affect the patient experience rating (overall satisfaction).

## Discussion

The survey provided exceptional data highlighting strengths and identifying previously unknown areas for improvement. Alberta retinal sub-specialty clinics had numerous strengths. Despite the high volume of patients seen each day at those clinics, most patients were seen by their retina sub-specialist within a reasonable amount of time. Patients reported that the on-call services were provided in a timely response (93%). Most patients also felt that their doctor spent enough time with them (90%) and listened to their concerns (90%). There was a high patient experience rating (overall satisfaction) of 4.63 out of 5. This is in line with the findings of Bahaziq and Crosby, Fuertes et al., and Bakar et al. who reported that patient satisfaction was associated with higher treatment adherence and better patient outcomes [5–7].

Areas in which our retinal specialists could improve their clinical care were also identified. More than half of the participants (57%) received intraocular injections for treatment of various conditions such as diabetic retinopathy, macular degeneration and retinal vein occlusions. However, more than half of the patients (51%) did not feel that the risks of the procedure were fully explained. These risks include pain, bleeding, retinal tears, cataract, infection, and vision loss. Such discussion is important for the patient’s decision making; it is also a critical aspect of the informed consent process. Physician-patient communication surrounding risks can increase patient satisfaction [13]. Failure to outline risks can also place a physician at risk of medico-legal liability [14]. Most patients did not know what intravitreal medications they were receiving (81%). Patients must understand the nature of their illness and comprehend available treatment options including what medication they are receiving.

Our findings are consistent with the findings of *Schoenfelder* et al and *Peterson* et al, who reported that time spent with the doctor, communication during the appointment, doctor’s kindness and accessibility were associated with higher patient satisfaction than other patient demographics; the latter reporting on glaucoma clinics [15, 16].

Improving practical patient encounter skills such as explaining procedure risks will need to be addressed. Follow up work will focus on strategies to improve the procedure’s consent process. The Kellogg Eye Center at the University of Michigan, for example, has established a quality improvement program which monitors physician error through records and surveys that are presented to physicians in peer environments with improvement monitoring [17]. A similar program might be established in our clinics to allow continuous quality improvement.

Patient wait time in clinic is an issue for most medical clinics. We were not surprised to see that shorter wait times were associated with higher levels of patient experience rating (overall satisfaction). Such issue could be addressed by reducing wait times or addressing patient’s perception of how long they wait. However, a study in Hamilton, Ontario found that telling patients anticipated wait time information did not affect their satisfaction level [18]. As such, working with staff to minimize wait times might have a greater impact. Indeed, Callaway et al. reported reduced wait times when OCT was decentralized to the clinic’s technician [19].. More research will need to be done to identify factors specific to each clinic surrounding wait times.

Our study is not without limitations. Convenient sampling could have introduced bias and affected the generalizability of the study findings. However, upon inspection, the characteristics of the participants corresponded closely to the characteristics of patients who attend the retina sub-specialty clinics. The data in the present study are self-reported; such data can be prone to social desirability bias.

Overall, we believe the results of this survey reflect positively on the clinical care provided by retina sub-specialists in Alberta. Understanding both the strengths and weaknesses of our practices is vital to the improvement of quality of care for patients. The next steps will be to implement methods to improve areas of weakness and while maintaining or improving clinic strengths as discussed in conjunction with the PLP. Details from this study have been provided to individual retina specialists/clinics for their own personal and collective improvement. This information should provide insights on gaps in the patient experience and allow the development of strategies to address patient concerns. These questions may be adapted to other clinics for future use.

## Conclusion

This is the first patient satisfaction survey assessing the quality of care found at office-based retina clinics in Canada. Data collected from our study will help retina specialists to improve eye care delivery for their patients.

## Data Availability

The datasets generated and analysed during the current study are not publicly available due to the specific and sensitive responses pertaining to each retinal physician involved but are available from the corresponding author on reasonable request.

## Abbreviations

OCT: Optical Coherence Tomography
PLP: Physician Learning Program
